# Misoprostol for medical treatment of missed abortion: a systematic review and network meta-analysis

**DOI:** 10.1038/s41598-017-01892-0

**Published:** 2017-05-10

**Authors:** Hang-lin Wu, Sheeba Marwah, Pei Wang, Qiu-meng Wang, Xiao-wen Chen

**Affiliations:** 1 Department of Obstetrics and Gynaecology, Hangzhou Women’s Hospital, Hangzhou, 310008 Zhejiang China; 2 Department of Obstetrics and Gynaecology, VMMC and Safdarjung Hospital, New Delhi, 110029 India

## Abstract

The efficacy and safety of misoprostol alone for missed abortion varied with different regimens. To evaluate existing evidence for the medical management of missed abortion using misoprostol, we undertook a comprehensive review and meta-analysis. The electronic literature search was conducted using PubMed, the Cochrane Library, Embase, EBSCOhost Online Research Databases, Springer Link, ScienceDirect, Web of Science, Ovid Medline and Google Scholar. 18 studies of 1802 participants were included in our analysis. Compared with vaginal misoprostol of 800 ug or sublingual misoprostol of 600 ug, lower-dose regimens (200 ug or 400 ug) by any route of administration tend to be significantly less effective in producing abortion within about 24 hours. In terms of efficacy, the most effective treatment was sublingual misoprostol of 600 ug and the least effective was oral misoprostol of 400 ug. In terms of tolerability, vaginal misoprostol of 400 ug was reported with fewer side effects and sublingual misoprostol of 600 ug was reported with more side effects. Misoprostol is a non-invasive, effective medical method for completion of abortion in missed abortion. Sublingual misoprostol of 600 ug or vaginal misoprostol of 800 ug may be a good choice for the first dose. The ideal dose and medication interval of misoprostol however needs to be further researched.

## Introduction

Missed abortion is defined as unrecognized intrauterine death of the embryo or fetus without expulsion of the products of conception. It constitutes approximately 15% of clinically diagnosed pregnancies^1^. Women experiencing a missed abortion may have no self-awareness due to the lack of obvious symptoms.

With around 95% success rate, surgical evacuation is regarded as the standard treatment for missed abortion, which had been widely performed all over the world in the past 50 years^2^. However, the costs of surgery and hospitalization, as well as the complications associated with surgery and anaesthesia are a major unresolved concern. Besides infection and bleeding, decreased fertility caused by intrauterine adhesions may be unacceptable for women with missed abortion, who have not yet fulfilled their motherhood desires. Some studies have thus suggested that expectant or medical management might be more suitable instead of surgical evacuation^3, 4^.

Expectant management has been reported with unpredictable success rate ranging from 25–76%^5–7^. Waiting for spontaneous expulsion of the products of conception would waste much time, during which women may suffer uncertainty and anxiety^5^. When additional surgical evacuation is needed owing to failure, they may suffer from an emotional breakdown. It is thus not recommended for missed early miscarriage due to the risks of emergency surgical treatment and blood transfusion^8^.

Misoprostol is a synthetic prostaglandin E1 analogue which was originally developed to prevent non-steroidal anti-inflammatory drugs related gastric ulcers. However it has been used for various other indications in obstetrics and gynaecology. Medical management using misoprostol or combined with mifepristone for missed abortion had been widely researched^9–13^. Some studies have reported that medical treatment with mifepristone and misoprostol in women with missed abortion would increase the incidence of excessive bleeding^11–13^. Apart from this, mifepristone is more expensive which will add to unnecessary expenses.

The efficacy and safety of misoprostol alone for missed abortion was established in many studies^14–17^. However, route of administration of misoprostol and success rates varied among the studies. It could be given by oral, sublingual or vaginal, while the doses ranged from 100 micrograms to 800 micrograms^14–19^. The most suitable route and dose of misoprostol for missed abortion is not yet clear. A single dose of 800 micrograms of misoprostol by vaginal or oral for missed abortion was recommended by National Institute for Health and Care Excellence (NICE)^20^. However some studies reported converse opinion, by pointing out that a lower dose or different routes of misoprostol may be equally effective^21, 22^.

So we evaluated the existing evidence for the medical management of missed abortion using misoprostol, with the hope of finding alternate suitable management strategies for surgical termination, which must be highly effective and with fewer side effects.

## Results

### Literature Search

**Figure 1:**
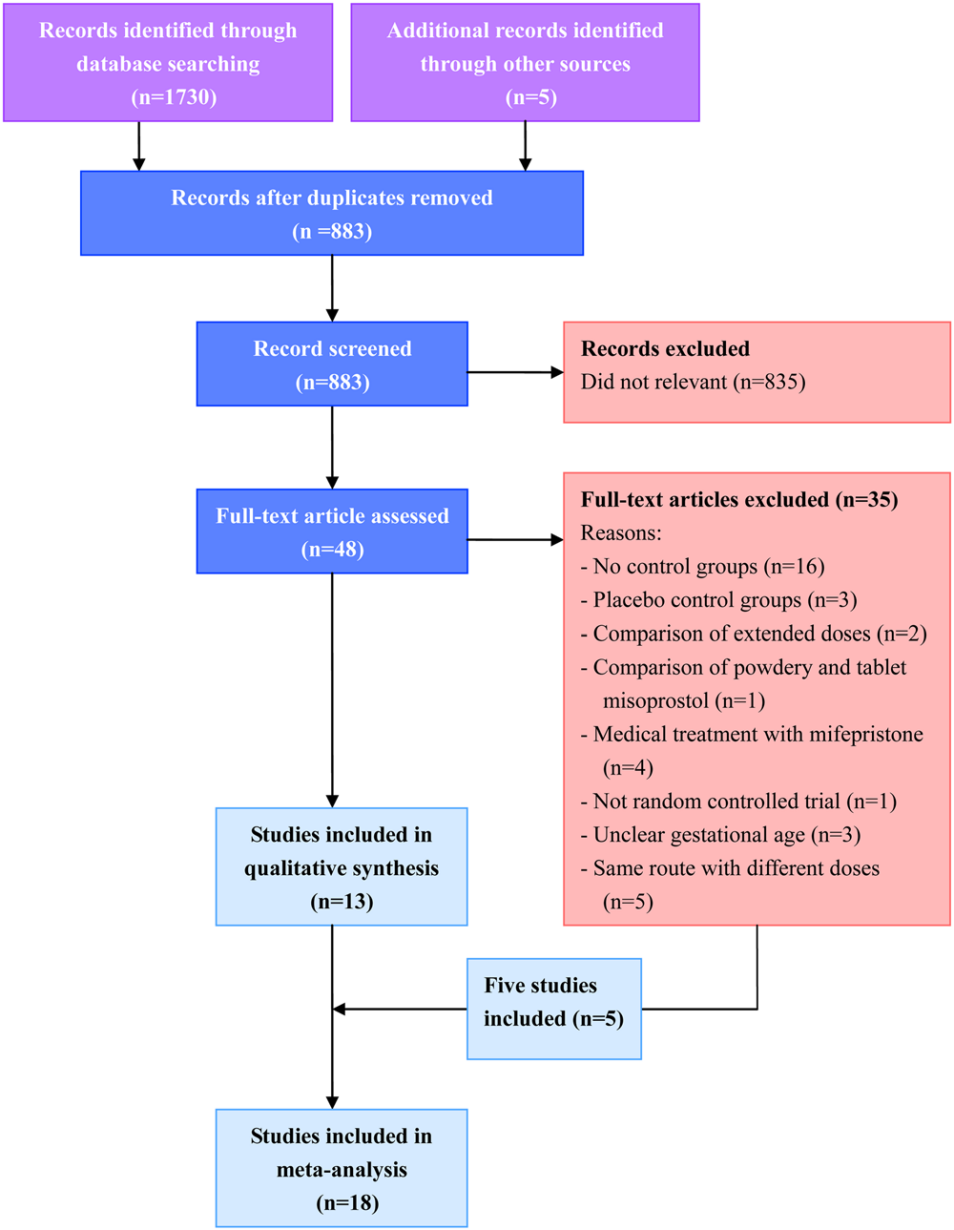
Article retrieval and screening.

**Figure 2:**
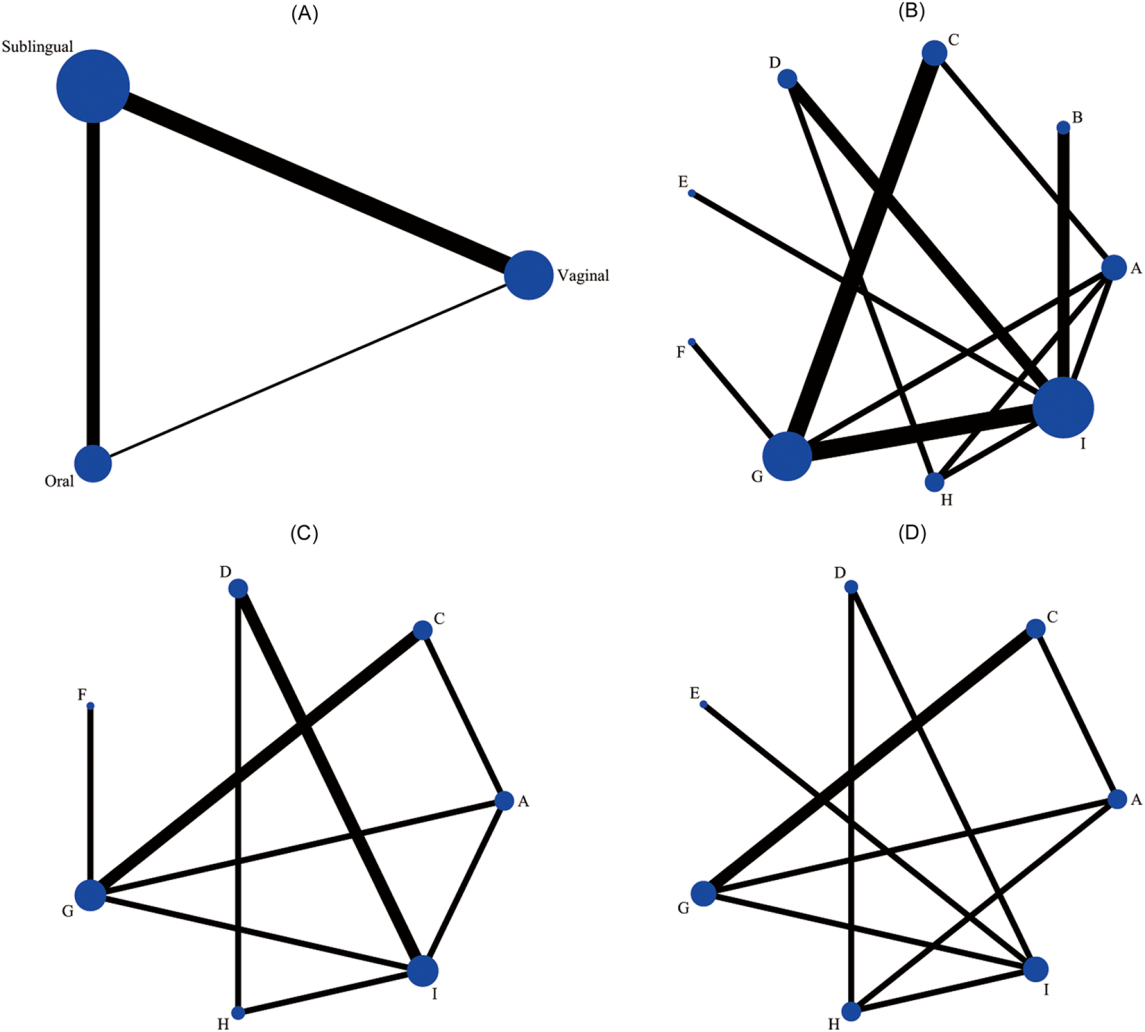
Network diagram of all studies and studies included in analyses of complete abortion rate within about 24 hours and main side effects. (**A**) Studies comparing different routes of misoprostol. (**B**) Studies comparing different routes or doses of misoprostol. (**C**) Complete abortion rate within about 24 hours. (**D**) Main side effects totally. Studies are classified according to the first dose of misoprostol in both groups; The width of the lines is proportional to the number of trials directly comparing each pair of interventions; The size of each node is proportional to the number of trails comparing a single intervention totally. Interventions are sequenced as follows: A. Oral 400 ug; B. Oral 800 ug; C. Sublingual 400 ug; D. Sublingual 600 ug; E. Sublingual 800 ug; F. Vaginal 200 ug; G. Vaginal 400 ug; H. Vaginal 600 ug; I. Vaginal 800 ug.

Overall, 1735 articles were identified by the search and 48 potentially eligible articles were retrieved in full text. Of these articles, 35 were excluded for reasons shown in Fig. 1. The remaining 13 articles met our predefined inclusion criteria. Most of studies compared vaginal route of misoprostol with sublingual or oral route while only one study compared sublingual route with oral route (Fig. 2A). When it turned to a network meta-analysis, another five articles which compared misoprostol with different doses in the same route were also included in our work. The network diagram of all included studies is shown in Fig. 2B.

### Study Characteristics

In all, 18 studies of 1802 participants, published between 1999 and 2016, were included in our analysis. The primary characteristics of the studies are tabulated in Supplementary Table S1. Most of the studies were form India and Thailand. The maximum gestational age of participants in all the studies ranged from 8 weeks to 13 weeks, except one study which reported the outcomes separately according to different trimesters^23^. Interventions in the groups varied in terms of routes, doses and medication intervals and we used the first dose to classify them. In most of studies, complete abortion was defined as complete expulsion of the products of conception without surgical intervention. However in four studies, a less than 15 mm intrauterine tissue diameter on ultrasound scan was taken as the cutof f ^24–27^, while one research adopted the criterion for endometrial thickness less than 10 mm^28^. Not all trials reported the same outcomes, especially for the follow-up time. A complete abortion rate within about 24 hours (24 to 28 hours) was mostly mentioned. We calculated it as our primary outcome. There was insufficient data to evaluate for complete abortion rate within 12 hours, 48 hours or 7 days.

For the reported side effects, we could only compare the incidence of nausea or vomiting, diarrhoea and fever. There was insufficient data to analyze other side effects. The mean time taken to abortion was difficult to evaluate due to different follow-up time. For each outcome we have indicated the number of trials contributing data to the network meta-analysis (Fig. 2C and D). Complete abortion rate within about 24 hours of any intervention and side effects of all the interventions in the analysis are presented in Supplementary Table S2.

Risk of bias was summarized in Supplementary Table S3. It was categorized the risk of bias as unclear when no related information reported could be used. Most of the included trials described adequate randomization processes; however most of them were assessed as having an unclear risk of bias for allocation concealment and blinding.

### Meta-analysis

**Figure 3:**
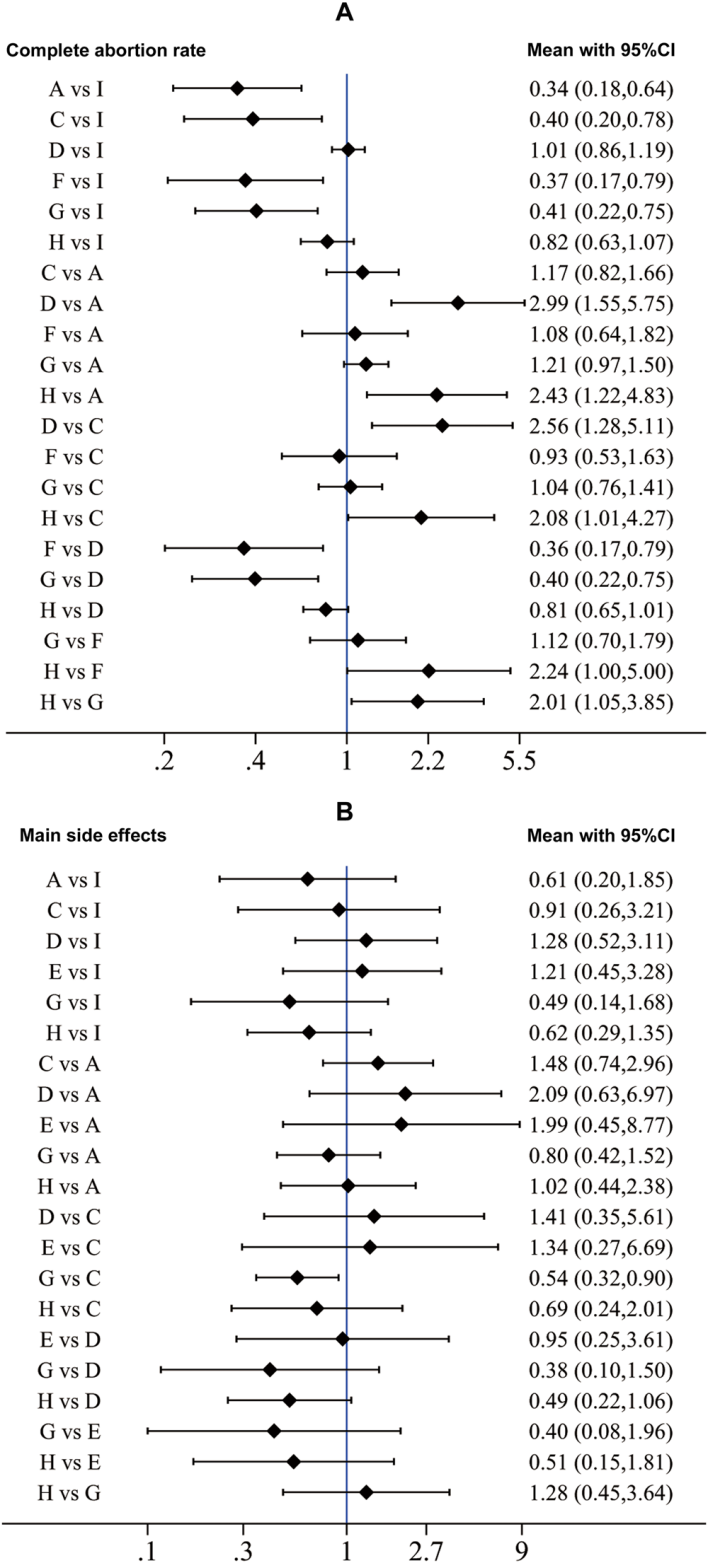
Network meta-analysis of complete abortion rate within about 24 hours and main side effects. Interventions are sequenced as follows: A. Oral 400 ug; C. Sublingual 400 ug; D. Sublingual 600 ug; E. Sublingual 800 ug; F. Vaginal 200 ug; G. Vaginal 400 ug; H. Vaginal 600 ug; I. Vaginal 800 ug.

The results of the network meta-analysis for the outcomes are presented as forest plots in Fig. 3. Compared with vaginal misoprostol of 800 ug, lower-dose regimens (200 ug or 400 ug) by any route of administration tend to be significantly less effective in producing abortion within about 24 hours. Similar results can be seen in another comparison with sublingual misoprostol of 600 ug. For the comparison between the two regimens, there is no significant difference (RR 1.01, 95% CI 0.86 to 1.19). For the same dose of 600 ug, administration by vaginal route seems to be less effective than sublingual route, however it is not significant (RR 0.81, 95% CI, 0.65 to 1.01). For vaginal misoprostol, doses of 600 ug and 800 ug have no significant differences in producing miscarriage within about 24 hours (RR 0.82, 95% CI 0.63 to 1.07). In the analysis of main side effects, significant difference could be seen only in the comparison of vaginal and sublingual misoprostol of 400 ug (RR 0.54, 95% CI 0.32 to 0.90). For the same dose of 600 ug, administration by vaginal route seems to accompany with fewer side effects than sublingual route, which is not significant again (RR 0.49, 95% CI, 0.22 to 1.06). In detailed comparison for nausea or vomiting, there were no significant differences observed amongst all the regimens (Supplementary Figure S1). For the incidence of diarrhoea, sublingual route seems to be more common than vaginal route with same doses of 600 ug and 400 ug (Supplementary Figure S2). For the incidence of fever, sublingual route also seems to be more common than vaginal or oral route with a same dose of 400 ug (Supplementary Figure S3).

Tests of consistency showed that there was no difference between the direct and indirect estimates in all close loops in the analysis of complete abortion rate (Supplementary Figure S4). The comparison-adjusted funnel plots of the network meta-analysis for complete abortion rate were not suggestive of any publication bias or small study effect (Supplementary Figure S5). The percentage contribution of each direct and indirect comparisons is presented as a table in Supplementary Figure S6. Inconsistency could be seen in a close loop (sublingual misoprostol of 600 ug, vaginal misoprostol of 600 ug and 800 ug) in the analysis of main side effects (Supplementary Figure S7). It was due to the inconsistency in the analysis of nausea or vomiting (Supplementary Figure S8). No publication bias or small study effect was found in the analysis of main side effects (Supplementary Figure S9).

The results of sensitivity analyses of complete abortion rate were shown in Supplementary Table S4. In the first sensitivity analysis we excluded one study in which gestational age of the participants was below 8 weeks while in the second we excluded another study in which complete abortion was defined as complete expulsion of the products of conception and endometrial thickness <10 mm. The results were robust for the two sensitivity analyses. When we excluded studies in which only single dose of misoprostol was used in both groups, pre-existed significantly differences disappeared and some of the confidence intervals were wide and across the null line. We reviewed studies related in the close loop with inconsistency, it was impossible to exclude any study for a reasonable argument. In the sensitivity analysis excluding studies with only single dose of misoprostol, the significant difference between vaginal and sublingual misoprostol of 400 ug still existed (Supplementary Table S5).

**Figure 4:**
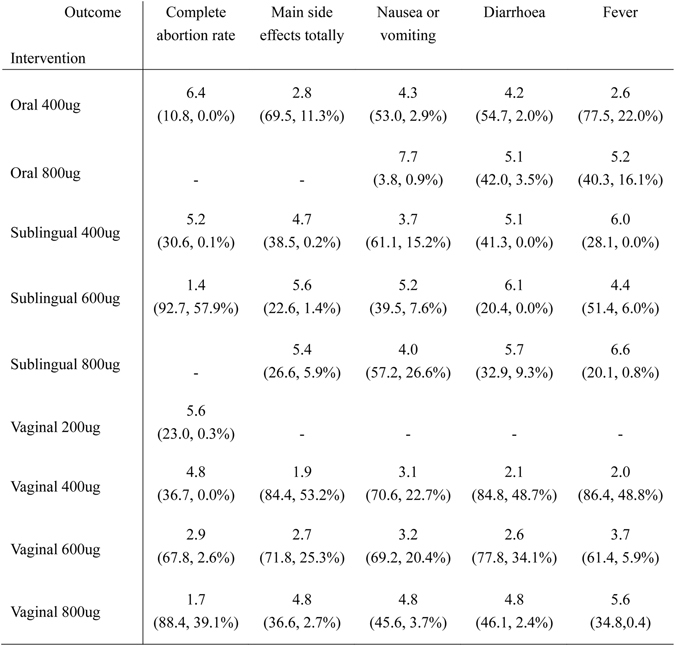
Ranking of all the interventions in network meta-analysis. Information of ranking is located at the intersection of the column-defining outcome and the row-defining intervention; The number in the first row is the ranking of all the interventions; The first number below in brackets is the surface under the cumulative ranking curve (SUCRA) while the second is the probability of the intervention to be the best.

The ranking of interventions based on cumulative probability plots and surfaces under the cumulative ranking curve (SUCRAs) is presented in Fig. 4. In terms of efficacy, the most effective treatment was sublingual misoprostol of 600 ug and the least effective was oral misoprostol of 400 ug. In terms of tolerability, vaginal misoprostol of 400 ug was reported with fewer side effects and sublingual misoprostol of 600 ug was reported with more side effects.

## Discussion

This network meta-analysis represents the most comprehensive synthesis of data for medical treatment using misoprostol for missed abortion. It was found that higher-dose regimens were associated with higher complete abortion rate and more sides effects. Sublingual misoprostol of 600 ug or vaginal misoprostol of 800 ug as the first dose was more effective in producing complete abortion within about 24 hours. However the superiority decreased with multiple doses. It could be explained that a single high-dose of misoprostol might have produced complete abortion in most of women^29–31^. If multiple doses were given, more women with lower-dose misoprostol would convert into complete abortion, which was confirmed by Kovavisarach^30^. We found that the least effective treatment was the oral misopristol of 400 ug. It was due to the liver first-pass effect which greatly reduced the bioavailability of the drug. Alternative routes of administration like vaginal and sublingual avoid the liver first-pass effect because they allow drugs to be absorbed directly into the systemic circulation.

Side effects were most likely to appear in sublingually or orally administered misoprostol. A low dose vaginal misoprostol was reported with the fewest side effects, accompanied by low complete abortion rate^32^. Compared with vaginally or orally administered misoprostol, sublingual misoprostol of 600 ug or 400 ug was associated with more frequent diarrhoea and fever. It was due to the pharmacokinetics of misoprostol, which showed that sublingual misoprostol had the shortest onset of action, the highest peak concentration and greatest bioavailability among the routes of administration^33^.

Vaginal misoprostol of 800 ug was recommend for missed abortion by National Institute for Health and Care Excellence(NICE) and some clinical guidelines^8, 20^. The results of our meta-analysis lead support to this regimen for medical treatment of missed abortion, however the question of whether sublingual misoprostol of 600 ug is better raises. Apart from this, the incidence of side effects reported was still higher than we expected for these regimens. A variety of methods were researched to increase the efficacy of misoprostol in order to reduce the dose. Some studies discussed the administration of different types of misoprostol, such as gel form and powder form, however the efficacy was not improved^34, 35^. Some studies discussed the efficacy of moistened misoprostol by acetic acid or normal saline, conclusion was made that vaginal misoprostol either moistened with normal saline or acetic acid was comparable in terms of efficacy and adverse effects^36–38^. Some studies reported different methods to combine misoprostol with laminaria tents or castor oil, however these studies did not focus on the efficacy of these methods to produce abortion for women with missed abortion^39, 40^.

Limited articles could be found about the efficacy and tolerability of sublingual or oral misoprostol of 800 ug which made us difficult to evaluate. Only in one study it was compared with vaginal misoprostol of 800 ug, the authors found that sublingual misoprostol was as effective as vaginal misoprostol and most side effects were similar in both groups, but heavy bleeding was more common in the sublingual group^41^. Two studies reported that oral misoprostol or vaginal of 800 ug was comparable in terms of efficacy while more side effects were reported in oral misoprostol of 800 ug in one study^42, 43^. Further research on the efficacy and tolerability of sublingual or oral misoprostol of 800 ug is needed. At present, these regimens should not be regarded as the first-line of medical treatment of missed abortion.

In our work, complete abortion rate was calculated within about 24 hours. Seldom studies reported complete abortion rate within longer follow-up time, they suggested follow-up care to be offered one week following drug administration to ensure the highest success rate^43–45^. Due to the limited amounts of studies, it is difficult to draw any conclusions. The security of waiting at home needs further researched, especially for the incidence of excessive bleeding. For women needed emergency operation, cervical ripening was prepared due to the medical treatment and it is convenient to perform dilatation and curettage^24, 26^.

Despite the foregoing advantages, serious consideration should be given to the contraindication before planning for medical treatment for women with missed abortion. A missed early miscarriage (<14 weeks of gestation) should be defined by ultrasound findings and suspected ectopic pregnancy must be excluded. Women with unstable hemodynamics, signs of pelvic infections or sepsis also need to be excluded. Detailed medical histories, including the distance between home and hospital, past medical history, previous surgical history, allergic history, medication history, should be recorded. Medical treatment can only be considered in women without following contraindications: known allergy to misoprostol, previous caesarean section, mitral stenosis, hypertension, glaucoma, bronchial asthma, use of non-steroidal drugs and remote areas without hospital around.

All women must be informed of the advantages and disadvantages of surgical and medical treatment. For women who choose medical treatment, hospitalization is not necessary, but the follow-up period will be more important. Pain killers and anti-emetics, such as paracetamol and metoclopramide, should be offered to them as needed^20^. All women should be advised to contact the doctor in case of heavy bleeding or signs of infection. A follow-up visit is recommended to perform within 2 weeks after treatment. Pregnancy test, physical examination of the uterus, and ultrasound should be performed to confirm the status of abortion. In the event of failure, surgical management maybe needed.

One of the strengths of our study is the inclusion of only randomized clinical trial data in a specific population (i.e., women with missed abortion of no more than 14 weeks of gestation). Our meta-analysis included all studies published so far on this topic and statistical tests showed no significant potential publication biases. The protocol of this review was registered on the International Prospective Register of Systematic Reviews before the selection of articles.

Limitations of this analysis are obvious. For a net-work meta-analysis, only 18 studies were included in this analysis which might affect the accuracy of the results. For this reason, comparisons could not be performed for some results. Most of the included studies were not double blind. This was therefore a considerable source of bias that may have affected treatment or performance of these women. We classified the interventions according to the first dose, however it is obvious that different max doses or medication intervals will affect the results.

The relation between max doses or medication intervals with complete abortion rate or side effects need to be further researched. Another remaining question is whether there are methods to reduce the incidence of side effects when treated with misoprostol. Further studies should focus on the quality of trails, especially for the blinding of participants and researchers.

In conclusion, misoprostol is a non-invasive, effective medical method for completion of abortion in missed abortion. Sublingual misoprostol of 600 ug or vaginal misoprostol of 800 ug may be a good choice for the first dose. The ideal dose and medication interval of misoprostol however needs to be further researched.

## Methods

### Search strategy and selection criteria

For this meta-analysis, we searched PubMed, the Cochrane Library, Embase, EBSCOhost Online Research Databases, Springer Link, ScienceDirect, Web of Science, Ovid Medline and Google Scholar for randomized controlled trials (RCTs) published from the date of database inception to August 15th, 2016, comparing different routes of administration of misoprostol in the medical management of missed abortion. We also searched some related journals. No language or publication type limits were applied. The reference lists of selected articles were hand searched to identify any relevant articles. Study authors were contacted to supplement incomplete reports of the original papers. Detailed search strategy can be found in Supplementary Table S6.

Considering the gestational weeks available for surgical evacuation, women with missed abortion of no more than 14 weeks of gestation who received misoprostol treatment were assessed for inclusion into our meta-analysis. Women with incomplete abortion, threatened abortion or excessive uterine bleeding were excluded. Studies involving medical management with both mifepristone and misoprostol were also excluded.

We considered complete abortion rate for our primary analyses. Complete abortion was defined as complete expulsion of the products of conception without surgical intervention. Our secondary outcome was the side effects of misoprostol reported. The mean induction-abortion time would be also analyzed, if applicable.

### Data extraction and quality assessment

Two researchers (H-L.W. and P.W.) performed their own search independently. Data extraction and check for accuracy were resolved by other two researchers (Q-M.W. and X.-W.C.). Duplicate or irrelevant articles were excluded by screening of titles and abstracts. All remaining articles were screened in full text. Relevant information from the included trials was extracted with a predefined data extraction sheet. All researchers assessed the risk of bias independently according to the Cochrane Handbook for Systematic Reviews of Interventions^46^. Specifically, attention was focused on seven domains, i.e., random sequence generation, allocation concealment blinding of participants and personnel, blinding of the outcome assessments, incomplete outcome data, selective reporting and other biases. The review authors’ judgments were categorized as low risk, high risk, or unclear risk of bias. We categorized the risk of bias as unclear when no reported information could be used. The article was reviewed and revised by another researcher (S.M.). Any discrepancies were resolved by discussion within the review team.

### Statistical analysis

This study was registered with PROSPERO, number CRD42016046221. The full dataset is available online. After screening of the articles, we found the interventions in included articles were so varied in both the routes and doses of misoprostol that we could not carry out a direct comparison. We chose to perform a network meta-analysis instead. The strategies for data synthesis remained unchanged and the predefined analysis of subgroups with different doses was cancelled.

This network meta-analysis used all the available evidence, both direct and indirect, to evaluate relative effects of different routes or doses of misoprostol^47, 48^. Statistical analysis was performed with STATA (version 12.0). We used a continuity correction for studies with no events by adding 0.5 to both the events count and the total sample size. We presented results as summary risk ratio (RR) for dichotomous data and the mean difference (MD) for continuous data, both with 95% confidence intervals (CIs). Inconsistency between direct and indirect sources of evidence was statistically assessed by calculation of the difference between direct and indirect estimates in all closed loops in the network. Random effects models were used to estimate the inconsistency. If there was no inconsistency between direct and indirect sources of evidence, fixed effects models would be used in further analysis, otherwise random effects models would still be used and sensitivity analyses would be performed to exclude studies with possibilities of causing bias in the close loops. A comparison-adjusted funnel plot was used to detect publication bias and small study effect. We estimated the ranking probabilities for all treatments of being at each possible rank for each intervention and the treatment hierarchy was summarized and presented as surface under the cumulative ranking curve^49^.

## Electronic supplementary material

Supplementary information

## References

[1] Wood SL, Brian PH (2002). Medical management of missed abortion: a randomized clinical trial. Obstet Gynecol..

[2] Joint study of Royal College of general practitioner and Royal College of obstetrician and gynaecologist. Induced abortion operations and their early sequelae. *J R Coll Gen Pract*. **35**, 175–180 (1985). PMC1960135 3989781

[3] Chia KV, Ogbo VI (2002). Medical termination of missed abortion. J Obstet Gynaecol..

[4] Petrou S, Trinder J, Brocklehurst P, Smith L (2006). Economic evaluation of alternative management methods of first-trimester miscarriage based on results from the MIST trial. BJOG..

[5] Jukovic D, Ross JA, Nicoladies KH (1998). Expectant management of missed miscarriage. Br J Obstet Gynaecol..

[6] Neilsen S, Hahlin M (1995). Expectant management of first-trimester spontaneous abortion. Lancet..

[7] Luise C (2002). Outcome of expectant management of spontaneous first trimester miscarriage: observational study. BMJ..

[8] Huchon C (2016). Pregnancy loss: French clinical practice guidelines. European Journal of Obstetrics & Gynecology and Reproductive Biology..

[9] Anderson J (2009). A randomised controlled trial of oral versus vaginal misoprostol for medical management of early fetal demise. International Journal of Gynecology and Obstetrics..

[10] Kushwah DS, Kushwah B, Salman MT, Verma VK (2011). Acceptability and safety profile of oral and sublingual misoprostol for uterine evacuation following early fetal demise. Indian J Pharmacol..

[11] Leladier C (1993). Mifepristone (RU 486) induces embryo expulsion in first trimester non-developing pregnancies: a prospective randomised trial. Hum Reprod..

[12] Gronlund A (2002). Management of missed abortion: comparison of medical treatment with either mifepristone misoprostol or misoprostol alone with surgical evacuation. A multi-center trial in Copenhagen county, Denmark. Acta Obstet Gynecol Scand..

[13] Nielsen S, Hahlin M, Platz-Christensen JJ (1997). Unsuccessful treatment of missed abortion with a combination of an antiprogesterone and a prostaglandine E1 analogue. Br J Obstet Gynaecol..

[14] Tasnee S, Gul MS, Navid S, Alam K (2014). Efficacy and safety of misoprostolin missed miscarriage in terms of blood loss. Rawal Medical Journal.

[15] Seyam YS, Flamerzi MA, Abdallah MM, Ahmed B (2007). Vaginal misoprostol in the management of first trimester non-viable pregnancy. Qatar Medical Journal..

[16] Poveda C (2001). Intrauterine misoprostol: a new high effective treatment of missed abortion. Fertility and Sterility..

[17] Sharma D, Singhal SR, Rani XX (2007). Sublingual misoprostol in management of missed abortion in India. Trop Doct..

[18] EI-Sokkary HH (2016). Comparison Between Sublingual and Vaginal Administration of Misoprostol in Management of Missed Abortion. J Obstet Gynaecol India..

[19] Haberal A, Celikkanat H, Batioglu S (1996). Oral misoprostol use in early complicated pregnancy. Adv Contracept..

[20] National Institute for Health and Care Excellence guidelines. Ectopic pregnancy and miscarriage: diagnosis and initial management. www.nice.org.uk/guidance/cg154 (2012). 31393678

[21] Prasartsakulchai C, Tannirandorn Y (2004). A comparison of vaginal misoprostol 800 microg versus 400 microg in early pregnancy failure: a randomized controlled trial. J Med Assoc Thai..

[22] Seervi N (2014). Comparison of different regimes of misoprostol for the termination of early pregnancy failure. Medical Journal Armed Forces India..

[23] Shah N, Azam SI, Khan NH (2010). Sublingual versus vaginal misoprostol in the management of missed miscarriage. J Pak Med Assoc..

[24] Marwah S (2016). A Comparative Study to Evaluate the Efficacy of Vaginal vs Oral Prostaglandin E1 Analogue (Misoprostol) in Management of First Trimester Missed Abortion. J Clin Diagn Res..

[25] Akanksha L, Ramanjeet K, Priya S (2016). A study to compare the clinical outcome of sublingual and vaginal misoprostol in the medical management. Int J Reprod Contracept Obstet Gynecol..

[26] Tanha FD, Feizi M, Shariat M (2010). Sublingual versus vaginal misoprostol for the management of missed abortion. J Obstet Gynaecol Res..

[27] Rita GS, Kumar S (2006). A randomised comparison of oral and vaginal misoprostol for medical management of first trimester missed abortion. JK Science..

[28] Ayudhaya OP, Herabutya Y, Chanrachakul B, Ayuthaya NI (2006). O-Prasertsawat,P. A comparison of the efficacy of sublingual and oral misoprostol 400 microgram in the management of early pregnancy failure: a randomized controlled trial. J Med Assoc Thai..

[29] Hombalegowda RB, Samapthkumar S, Vana H, Jogi P, Ramaiah R (2015). A randomized controlled trial comparing different doses of intravaginal misoprostol for early pregnancy failure. Contraception..

[30] Srikhao N, Tannirandorn Y (2005). A comparison of vaginal misoprostol 800 microg versus 400 microg for anembryonic pregnancy: a randomized comparative trial. J Med Assoc Thai..

[31] Kovavisarach E, Jamnansiri C (2005). Intravaginal misoprostol 600 microg and 800 microg for the treatment of early pregnancy failure. Int J Gynaecol Obstet..

[32] Suchonwanit P (1999). Comparative study between vaginal misoprostol 200 mg and 400 mg in first trimester intrauterine fetal death and anembryonic gestation. Thai Journal of Obstetrics and Gynaecology..

[33] Tang OS, Schweer H, Seyberth HW, Lee SW, Ho PC (2002). Pharmacokinetics of different routes of administration of misoprostol. Hum Reprod..

[34] Pongsatha S, Tongsong T (2008). Randomized comparison of dry tablet insertion versus gel form of vaginal misoprostol for second trimester pregnancy termination. J Obstet Gynaecol Res..

[35] Saichua C, Phupong V (2009). A randomized controlled trial comparing powdery sublingual misoprostol and sublingual misoprostol tablet for management of embryonic death or anembryonic pregnancy. Arch Gynecol Obstet..

[36] Bhattacharjee N (2012). A randomized comparative study on vaginal administration of acetic acid-moistened versus dry misoprostol for mid-trimester pregnancy termination. Arch Gynecol Obstet..

[37] Pongsatha S, Tongsong T (2011). Randomized controlled study comparing misoprostol moistened with normal saline and with acetic acid for second-trimester pregnancy termination. Is it different?. J Obstet Gynaecol Res..

[38] Creinin MD, Carbonell JL, Schwartz JL, Varela L, Tanda R (1999). A randomized trial of the effect of moistening misoprostol before vaginal administration when used with methotrexate for abortion. Contraception..

[39] Jain JK, Mishell DR (1996). A comparison of misoprostol with and without laminaria tents for induction of second-trimester abortion. Am J Obstet Gynecol..

[40] Jmylyan M, Hydry M (2015). Comparison of the Efficacy of Castor Oil and Vaginal Misoprostol With Vaginal Misopostol Alone for Treatment of Missed Abortion. Arak Medical University Journal..

[41] Areerat S, Teerapat C (2014). Comparison of sublingual and vaginal misoprostol for termination of early pregnancy failure. Thai J Obstet Gynaecol..

[42] Mohammed, S. Oral versus vaginal misoprostol for termination of frist trimester missed abortion. *MSc for Cairo University*. http://www.erepository.cu.edu.eg/index.p-hp/cuth eses/thesis/view/13990 (2013).

[43] Ngoc NT, Blum J, Westheimer E, Quan TT, Winikoff B (2004). Medical treatment of missed abortion using misoprostol. Int J Gynaecol Obstet..

[44] Tang OS, Lau WN, Ng EH, Lee SW, Ho PC (2013). A prospective randomized repeated doses of vaginal study to compare the use of with sublingual misoprostol in the management of first trimester silent miscarriages. Hum Reprod..

[45] Tang OS (2006). A randomized trial to compare the use of sublingual misoprostol with or without an additional 1 week course for the management of first trimester silent miscarriage. Hum Reprod..

[46] Higgins, J. G. S. Cochrane Handbook for Systematic reviews of Interventions (v5.1.0). http://handbook.cochrane.org/ (2011).

[47] Caldwell DM, Ades AE, Higgins JP (2005). Simultaneous comparison of multiple treatments: combining direct and indirect evidence. BMJ..

[48] Dias S, Ades A, Sutton A, Welton N (2013). Evidence synthesis for decision making 2: a generalized linear modeling framework for pairwise and network meta-analysis of randomized controlled trials. Med Decis Making..

[49] Salanti G, Ades AE, Ioannidis JP (2011). Graphical methods and numerical summaries for presenting results from multiple-treatment meta-analysis: an overview and tutorial. J Clin Epidemiol..

