# Supplementary material for: Misoprostol for medical treatment of missed abortion: a systematic review and network meta-analysis
Source: Sci Rep. 2017 May 10;7:1664. doi: 10.1038/s41598-017-01892-0 (PMC5431938; doi:10.1038/s41598-017-01892-0)
Supplement: Supplementary file 1 — Supplementary information [file 41598_2017_1892_MOESM1_ESM.pdf]

Misoprostol for medical treatment of missed abortion: a systematic review and network meta-analysis

Hang-lin Wu[1*], Sheeba Marwah[2], Pei Wang[1], Qiu-meng Wang[1],Xiao-wen Chen[1]

[1] Department of Obstetrics and Gynecology, Hangzhou Women's Hospital, Hangzhou, Zhejiang, China. [2] Department of Obstetrics and Gynecology, VMMC and Safdarjung Hospital, New Delhi, India. Correspondence and requests for materials should be addressed to H.L.W. (email: hanglinwu@gmail.com)

Supplementary Table S1. Characteristics of the trials identified and included in the network meta-analysis

Author	Country	NO.of women	Maximum gestational age	Interventions		Outcomes
				Group A	Group B	
Marwah et al. (2016)	India	100	12 weeks	Oral<br>400ug misoprostol,<br>repeat every 6h,<br>maximum 3 doses	Vaginal<br>400ug misoprostol,<br>repeat every 6h,<br>maximum 2 doses	CAR within 24h;<br>Time to abortion;<br>Side effects;<br>Excessive bleeding;<br>Cervical ripening;<br>Satisfaction
Akanksha et al.(2016)	India	50	12 weeks	Sublingual<br>400ug misoprostol,<br>repeat every 4h,<br>maximum 5 doses	Vaginal<br>400ug misoprostol,<br>repeat every 4h,<br>maximum 5 doses	CAR within 28h;<br>Time to abortion;<br>Mean doses used;<br>Side effects;<br>Excessive bleeding
Seervi et al. (2014)	India	110	12 weeks	Sublingual<br>600ug misoprostol,<br>repeat every 6h,<br>maximum 3 doses	Vaginal<br>800ug misoprostol,<br>repeat every 6h,<br>maximum 3 doses	CAR within 24h;<br>Time to abortion;<br>Mean doses used;<br>Side effects;<br>Satisfaction
Latif et al. (2014)	Pakistan	60	13 weeks	Sublingual<br>600ug misoprostol,<br>repeat every 6h,<br>maximum 3 doses	Vaginal<br>800ug misoprostol and saline solution,<br>repeat every 6h,<br>maximum 3 doses	CAR within 26h;<br>Time to abortion;<br>Mean doses used;<br>Side effects;<br>Excessive bleeding

Sonsanoh et al.(2014)	Thailand	120	12 weeks	Sublingual 800ug misoprostol, repeat every 6h, maximum 3 doses	Vaginal 800ug misoprostol and saline solution, repeat every 6h, maximum 3 doses	CAR within 45h; Time to abortion; Mean doses used; Side effects; Satisfaction
Sobhy (2013)	Egypt	80	12 weeks	Oral 800ug misoprostol, repeat 400mg every 6h, maximum 2 doses	Vaginal 800ug misoprostol, repeat 400mg every 6h, maximum 2 doses	CAR(unclear time); Mean doses used; Side effects
Tanha et al. (2010)	Iran	220	13 weeks	Sublingual 400ug misoprostol, repeat every 6h, maximum unclear	Vaginal 400ug misoprostol, repeat every 6h, maximum unclear	CAR within 48h; Time to abortion; Mean doses used; Side effects; Satisfaction
Shah et al. (2010)	Pakistan	50	20 weeks	Sublingual 400ug misoprostol, repeat every 3h, maximum 5 doses	Vaginal 400ug misoprostol, repeat every 3h, maximum 5 doses	CAR within 28h;
Rita et al. (2006)	India	100	13 weeks	Oral 400ug misoprostol, repeat every 4h, maximum 3 doses	Vaginal 600ug misoprostol, repeat every 4h, maximum 2 doses	CAR within 12h; Time to abortion; Mean doses used; Side effects; Cervical ripening
Ayudhaya et al. (2006)	Thailand	138	12 weeks	Oral 400ug misoprostol, repeat every 4h, maximum 6 doses	Sublingual 400ug misoprostol, repeat every 4h, maximum 6 doses	CAR within 24h; Time to abortion; Side effects
Ngoc et al. (2004)	Vietnam	200	12 weeks	Oral 800ug misoprostol	Vaginal 400ug misoprostol	CAR within 48h; CAR within 7 days Time to abortion; Side effects
Tang et al. (2003)	China	80	13 weeks	Sublingual 600ug misoprostol, repeat every 3h, maximum 3 doses	Vaginal 600ug misoprostol, repeat every 3h, maximum 3 doses	CAR within 24h; CAR within 7 days Time to abortion; Side effects
Creinin et al. (1997)	America	18	8 weeks	Oral 400ug misoprostol, repeat every 24h, maximum 2 doses	Vaginal 800ug misoprostol, repeat every 24h, maximum 2 doses	CAR within 24h; CAR within 48h; Mean doses used; Side effects
Hombalegowda et al.(2015)	India	50	12 weeks	Vaginal 400ug misoprostol	Vaginal 800ug misoprostol	CAR within 24h; CAR within 48h; Time to abortion

Srikhao et al. (2005)	Thailand	50	12 weeks	Vaginal 400ug misoprostol	Vaginal 800ug misoprostol	CAR within 12h; CAR within 48h
Kovavisarach et al.(2005)	Thailand	114	12 weeks	Vaginal 600ug misoprostol	Vaginal 800ug misoprostol	CAR within 24h; Time to abortion; Side effects
Prasartsakulchai et al.(2004)	Thailand	50	12 weeks	Vaginal 800ug misoprostol	Vaginal 400ug misoprostol	CAR within 48h; Time to abortion; Side effects; Excessive bleeding
Suchonwanit (1999)	Thailand	212	12 weeks	Vaginal 200ug misoprostol repeat every 12h, maximum 3 doses	Vaginal 400ug misoprostol repeat every 12h, maximum 3 doses	CAR within 24h

Abbreviations: CAR, Complete abortion rate.

Intervention	Oral 400ug	Oral 800ug	Sublingual 400ug	Sublingual 600ug	Sublingual 800ug	Vaginal 200ug	Vaginal 400ug	Vaginal 600ug	Vaginal 800ug
Complete abortion rate	55/128	-	43/117	107/126	-	26/106	108/225	56/97	130/174
Nausea or vomiting	285/1135								
Diarrhea	284/1352								
Fever	161/1112								

Supplementary Table S2. Complete abortion rate of any intervention and side effects of all the interventions in the meta-analysis

Supplementary Table S3. Risk of bias in the included studies.

**Authors and publication year**	**Random sequence generation**	**Allocation concealment**	**Blinding of participants and personnel**	**Blinding of outcome assessor**	**Incomplete outcome data**	**Selective outcome reporting**
Marwah et al. (2016)	L	U	U	U	L	L

Akanksha et al.(2016)	L	U	U	U	L	L
Seervi et al. (2014)	L	U	U	U	L	L
Latif et al. (2014)	U	U	U	U	L	L
Sonsanoh et al.(2014)	L	L	U	U	L	L
Sobhy (2013)	L	L	U	U	L	L
Tanha et al. (2010)	L	H	H	U	L	L
Shah et al. (2010)	L	L	U	U	L	L
Rita et al. (2006)	L	U	U	U	L	L
Ayudhaya et al. (2006)	L	U	U	U	L	L
Ngoc et al. (2004)	L	U	H	U	L	L
Tang et al. (2003)	L	U	U	U	L	L
Creinin et al. (1997)	L	U	U	U	L	L
Hombalegowda et al.(2015)	U	U	U	U	U	U
Srikhao et al. (2005)	U	U	U	U	U	U
Kovavisarach et al.(2005)	L	L	L	L	L	L
Prasartsakulchai et al.(2004)	L	U	U	U	L	L
Suchonwanit (1999)	U	U	U	U	U	U

Abbreviations:L,low risk;H,high risk;U,unclear risk.

Supplementary Table S4. Sensitivity analyses of complete abortion rate(risk ratio and 95% CI)

Comparisons	All studies	Sensitivity analysis 1#	Sensitivity analysis 2*	Sensitivity analysis 3$
A vs. I	0.34(0.18,0.64)	0.37(0.19,0.73)	0.33(0.17,0.62)	0.16(0.02,1.11)
C vs. I	0.40(0.20,0.78)	0.43(0.21,0.87)	0.44(0.22,0.88)	0.19(0.03,1.33)
D vs. I	1.01(0.86,1.19)	1.01(0.86,1.20)	1.02(0.87,1.19)	1.05(0.93,1.18)
F vs. I	0.37(0.17,0.79)	0.40(0.18,0.88)	0.37(0.17,0.79)	0.17(0.02,1.28)
G vs. I	0.41(0.22,0.75)	0.44(0.24,0.84)	0.41(0.23,0.75)	0.19(0.03,1.35)
H vs. I	0.82(0.63,1.07)	0.82(0.63,1.07)	0.83(0.64,1.07)	0.95(0.74,1.23)
C vs. A	1.17(0.82,1.66)	1.16(0.80,1.66)	1.34(0.89,2.02)	1.17(0.84,1.62)
D vs. A	2.99(1.55,5.75)	2.72(1.34,5.52)	3.09(1.62,5.89)	6.56(0.95,45.58)
F vs. A	1.08(0.64,1.82)	1.07(0.63,1.82)	1.12(0.67,1.88)	1.08(0.66,1.77)
G vs. A	1.21(0.97,1.50)	1.19(0.94,1.51)	1.25(1.01,1.55)	1.20(1.01,1.45)
H vs. A	2.43(1.22,4.83)	2.21(1.05,4.64)	2.52(1.28,4.93)	5.96(0.85,41.91)
D vs. C	2.56(1.28,5.11)	2.35(1.14,4.87)	2.31(1.13,4.70)	5.60(0.79,39.93)
F vs. C	0.93(0.53,1.63)	0.93(0.52,1.64)	0.84(0.47,1.51)	0.93(0.54,1.60)
G vs. C	1.04(0.76,1.41)	1.03(0.75,1.42)	0.94(0.66,1.33)	1.03(0.76,1.39)
H vs. C	2.08(1.01,4.27)	1.91(0.90,4.07)	1.88(0.90.3.93)	5.09(0.70,36.79)
F vs. D	0.36(0.17,0.79)	0.39(0.17,0.89)	0.36(0.17,0.79)	0.17(0.02,1.22)
G vs. D	0.40(0.22,0.75)	0.44(0.23,0.85)	0.41(0.22,0.75)	0.18(0.03,1.28)
H vs. D	0.81(0.65,1.01)	0.81(0.65,1.02)	0.81(0.66,1.01)	0.91(0.72,1.14)
G vs. F	1.12(0.70,1.79)	1.12(0.69,1.79)	1.12(0.70,1.78)	1.11(0.71,1.75)
H vs. F	2.24(1.00,5.00)	2.06(0.89,4.76)	2.24(1.00,4.99)	5.50(0.74,41.09)
H vs. G	2.01(1.05,3.85)	1.85(0.93,3.68)	2.01(1.05,3.84)	4.93(0.70,34.96)

Interventions are sequenced as follows: A. Oral 400ug，C. Sublingual 400ug，D. Sublingual 600ug，F. Vaginal 200ug，G. Vaginal 400ug，H. Vaginal 600ug，I. Vaginal 800ug.

excluding one study in which gestational age of the participants was below 8 weeks; *excluding one study in which complete abortion was defined as complete expulsion of the products of conception and endometrial thickness <10mm; $excluding studies in which only single dose of misoprostol was used in both groups.

Supplementary Table S5. Sensitivity analysis of main side effects(risk ratio and 95% CI)

Comparisons	All studies	Sensitivity analysis*
A vs. I	0.61(0.20,1.85)	2.22(0.58,8.33)
C vs. I	0.91(0.26,3.21)	3.57(0.84,14.29)

D vs. I	1.28(0.52,3.11)	2.78(0.96,8.33)
E vs. I	1.21(0.45,3.28)	1.22(0.54,2.70)
G vs. I	0.49(0.14,1.68)	1.92(0.47,8.33)
H vs. I	0.62(0.29,1.35)	1.85(0.57,6.25)
C vs. A	1.48(0.74,2.96)	1.59(0.95,2.67)
D vs. A	2.09(0.63,6.97)	1.27(0.57,2.82)
E vs. A	1.99(0.45,8.77)	0.55(0.12,2.61)
G vs. A	0.80(0.42,1.52)	0.88(0.54,1.41)
H vs. A	1.02(0.44,2.38)	0.84(0.45,1.58)
D vs. C	1.41(0.35,5.61)	0.80(0.31,2.07)
E vs. C	1.34(0.27,6.69)	0.34(0.07,1.78)
G vs. C	0.54(0.32,0.90)	0.55(0.38,0.79)
H vs. C	0.69(0.24,2.01)	0.53(0.23,1.20)
E vs. D	0.95(0.25,3.61)	0.43(0.11,1.65)
G vs. D	0.38(0.10,1.50)	0.69(0.27,1.75)
H vs. D	0.49(0.22,1.06)	0.67(0.40,1.10)
G vs. E	0.40(0.08,1.96)	1.59(0.31,8.33)
H vs. E	0.51(0.15,1.81)	1.54(0.37,6.40)
H vs. G	1.28(0.45,3.64)	1.04(0.47,2.27)

Interventions are sequenced as follows: A. Oral 400ug，C. Sublingual 400ug，D. Sublingual 600ug，E. Sublingual 800ug，G. Vaginal 400ug，H. Vaginal 600ug，I. Vaginal 800ug.
*excluding study in which only single dose of misoprostol was used in both groups.

Supplementary Table S6. Detailed search strategy for systematic review

Databases searched	PubMed, the Cochrane Library, Embase, EBSCOhost Online Research Databases, Springer Link, Sciencedirect, Web of Science and Ovid Medline and Google Scholar.
Search strategy for Pubmed(similar strategies were applied to other databased)	(((((((((((((Misoprostol) OR Novo-Misoprostol) OR SC-29333) OR SC 29333) OR SC29333) OR SC-30249) OR SC 30249) OR SC30249) OR Apo-Misoprostol) OR Glefos) OR Cytotec)) AND ((((pregnancy failure[Title/Abstract]) OR "Abortion, Missed"[Mesh]) OR ((((missed) OR silent)) AND ((Abortion*[Title/Abstract]) OR miscarriage*[Title/Abstract]))) OR ((fetal[Title/Abstract]) AND (((demise[Title/Abstract]) OR death[Title/Abstract]) OR deaths[Title/Abstract]))))) AND ("randomized controlled trial"[pt] OR "controlled clinical trial"[pt] OR randomized[tiab] OR placebo[tiab] OR "drug therapy"[sh] OR randomly[tiab] OR trial[tiab] OR groups[tiab])
Other sources	Some related journals were also searched. The reference lists of selected articles and reviews were hand searched to identify any relevant articles.

Supplementary Figure S1. Comparison of the incidence of nausea or vomiting

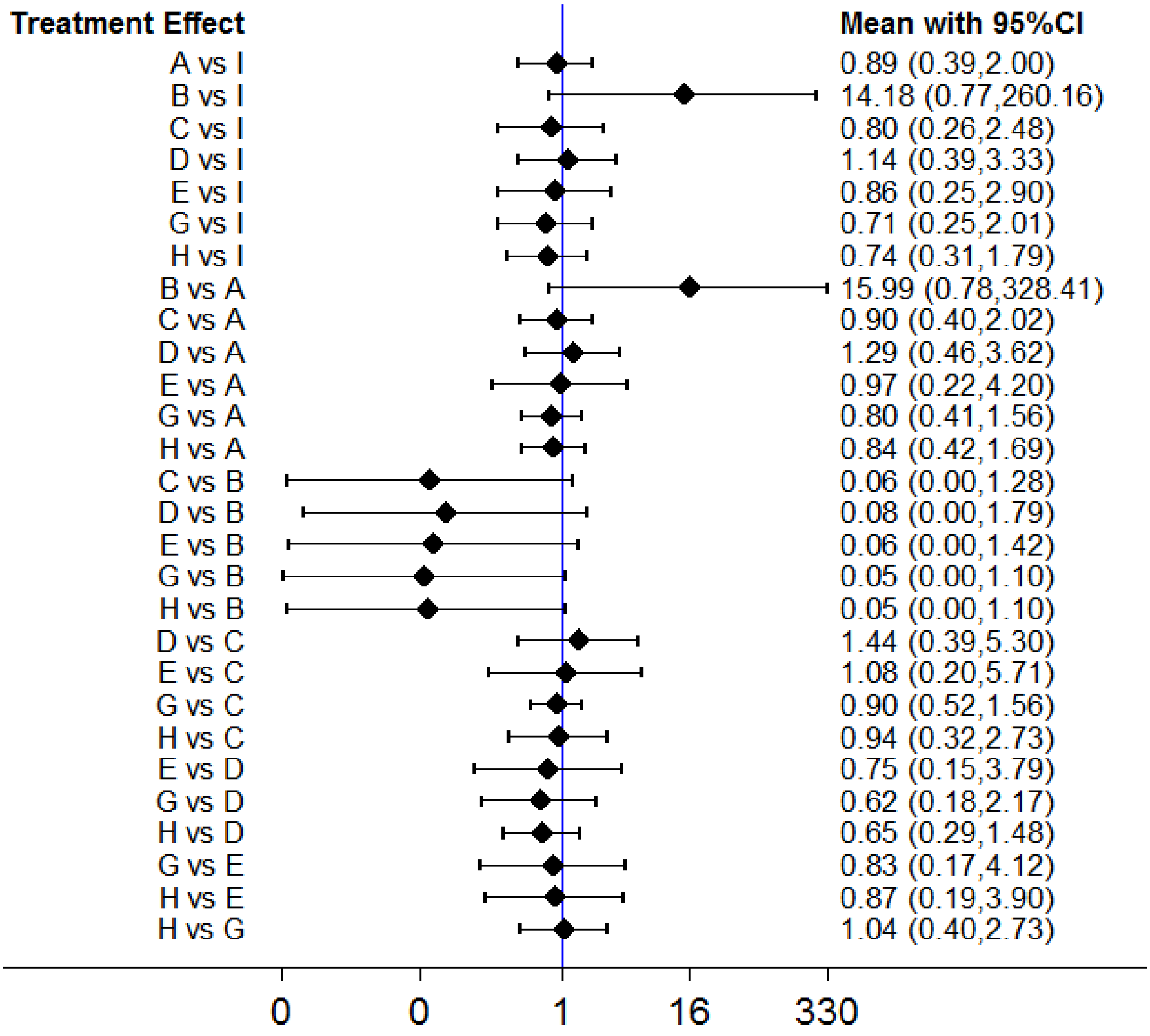

Interventions are sequenced as follows: A. Oral 400ug , B. Oral 800ug , C. Sublingual 400ug , D. Sublingual 600ug , E. Sublingual 800ug , G. Vaginal 400ug , H. Vaginal 600ug , I. Vaginal 800ug.

Supplementary Figure S2. Comparison of the incidence of diarrhoea

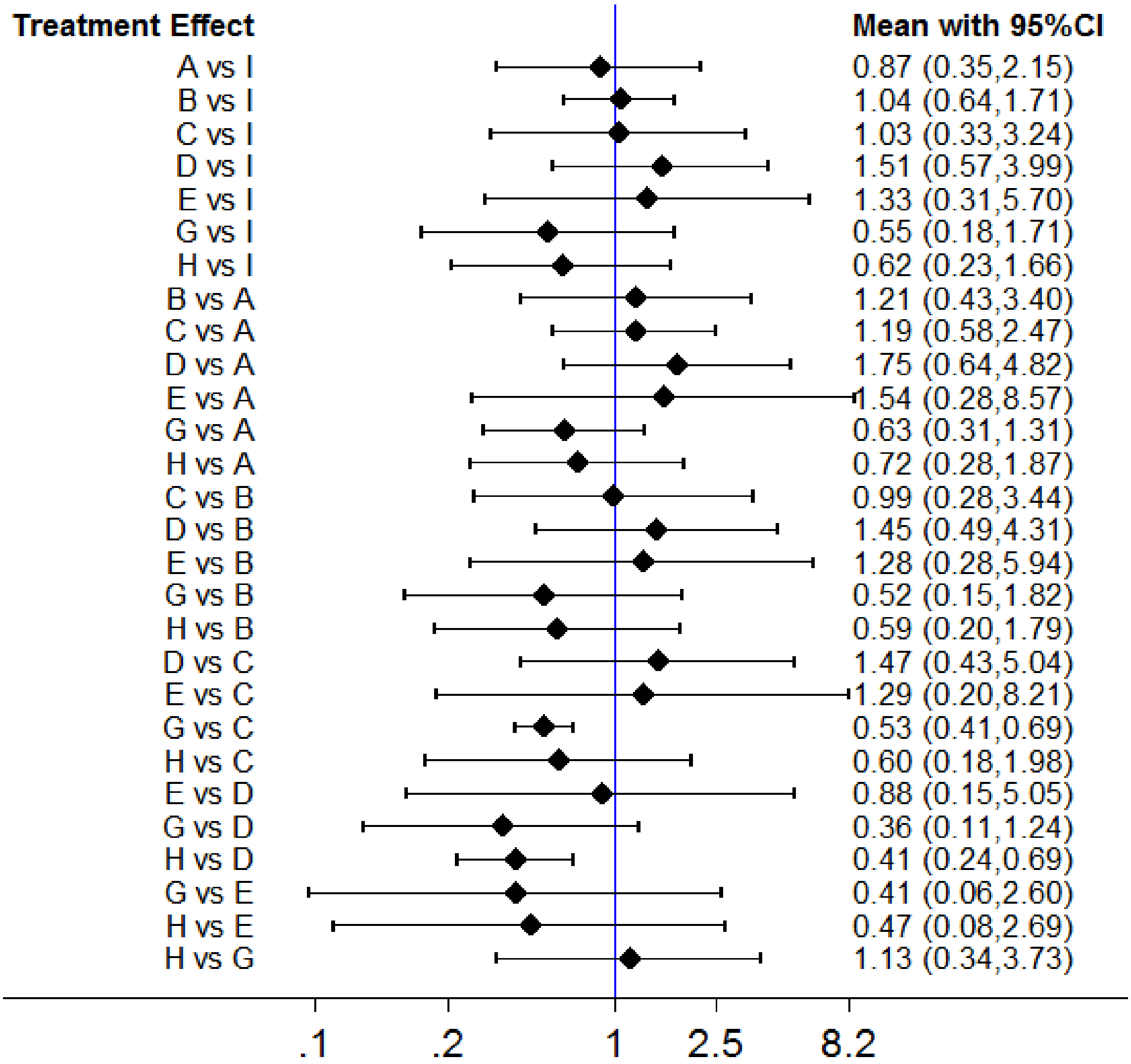

Interventions are sequenced as follows: A. Oral 400ug , B. Oral 800ug , C. Sublingual 400ug , D. Sublingual 600ug , E. Sublingual 800ug , G. Vaginal 400ug , H. Vaginal 600ug , I. Vaginal 800ug.

Supplementary Figure S3. Comparison of the incidence of fever

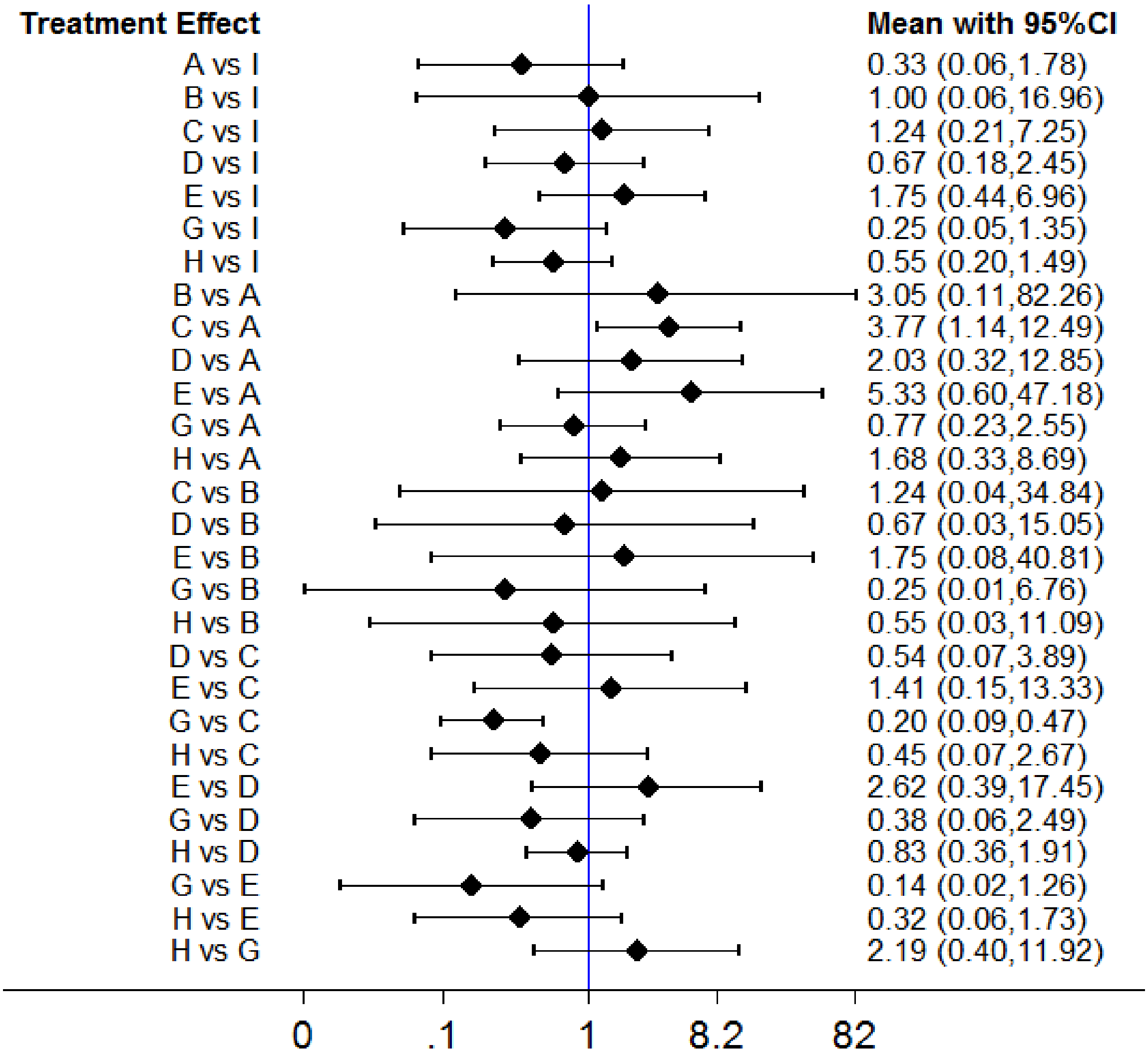

Interventions are sequenced as follows: A. Oral 400ug , C. Sublingual 400ug , D. Sublingual 600ug , E. Sublingual 800ug , F. Vaginal 200ug , G. Vaginal 400ug , H. Vaginal 600ug , I. Vaginal 800ug.

Supplementary Figure S4.Calculation of the difference between direct and indirect estimates in all closed loops in the analysis of complete abortion rate

Loop	IF	95%CI (truncated)	Loop-specific Heterogeneity(τ^2)
A-G-I	0.80	(0.00,2.84)	0.000
A-C-G	0.44	(0.00,1.16)	0.000
D-H-I	0.36	(0.00,0.78)	0.000

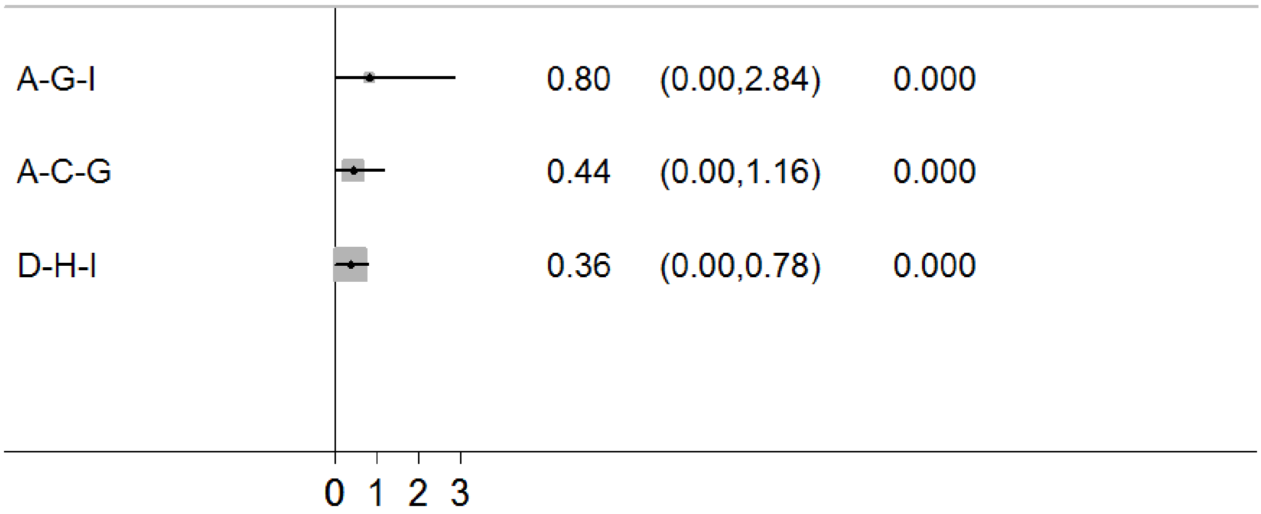

Abbreviations: IF, inconsistency factors.

Interventions are sequenced as follows: A. Oral 400ug，C. Sublingual 400ug，D. Sublingual 600ug，G. Vaginal 400ug，H. Vaginal 600ug，I. Vaginal 800ug.

Supplementary Figure S5. Comparison-adjusted funnel plot in the analysis of complete abortion rate

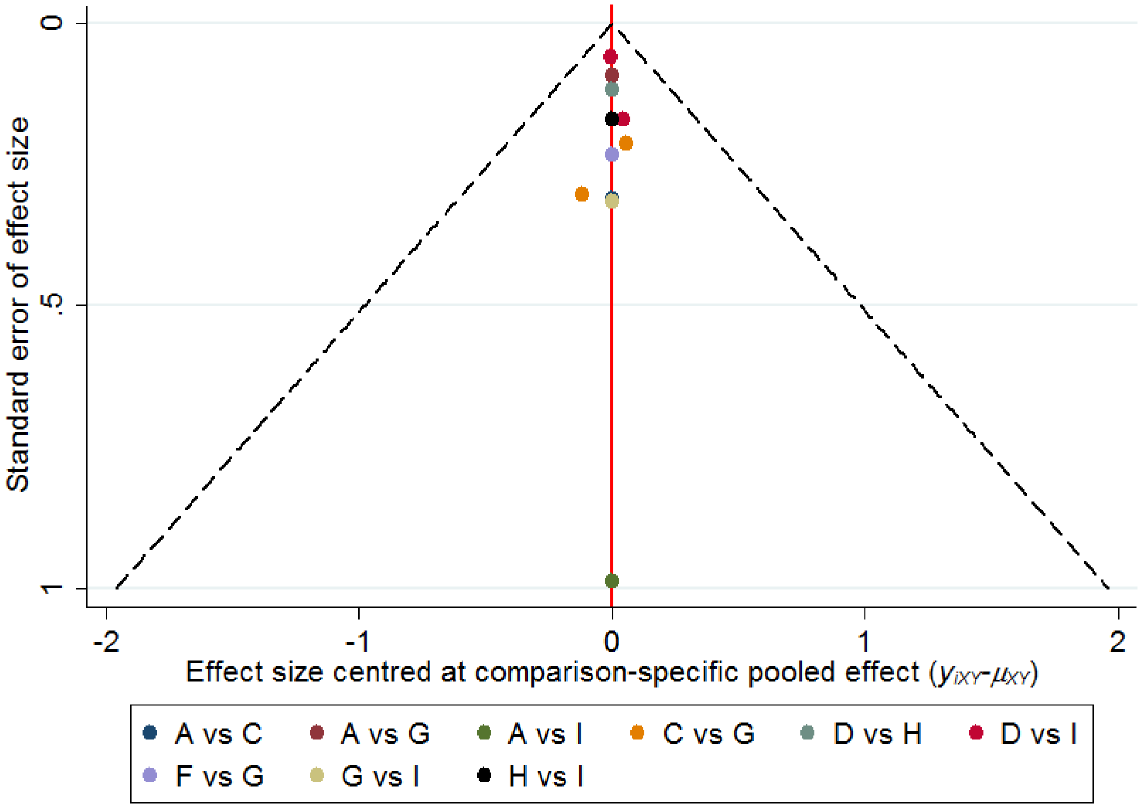

Interventions are sequenced as follows: A. Oral 400ug，C. Sublingual 400ug，D. Sublingual 600ug，F. Vaginal 200ug，G. Vaginal 400ug，H. Vaginal 600ug，I. Vaginal 800ug.

Supplementary Figure S6. Percentage contribution of each direct and indirect comparisons in the analysis of complete abortion rate

Network meta-analysis estimates	AvsC	AvsG	AvsI	CvsG	DvsH	DvsI	FvsG	GvsI	HvsI
	**Direct comparisons in the network**								
Mixed estimates									
AvsC	16.8	41.1	0.3	41.4	.	.	.	0.3	.
AvsG	6.0	86.6	0.7	6.0	.	.	.	0.7	.
AvsI	3.0	43.0	5.1	3.0	.	.	.	45.9	.
CvsG	18.3	18.2	0.1	63.2	.	.	.	0.1	.
DvsH	.	.	.	.	54.2	22.9	.	.	22.9
DvsI	.	.	.	.	6.6	86.7	.	.	6.6
FvsG	.	.	.	.	.	.	100.0	.	.
GvsI	0.5	7.9	8.4	0.5	.	.	.	82.6	.
HvsI	.	.	.	.	38.7	38.7	.	.	22.6
Indirect estimates									
AvsD	1.9	27.8	3.3	1.9	2.4	30.7	.	29.7	2.4
AvsF	3.1	44.8	0.4	3.1	.	.	48.3	0.4	.
AvsH	1.6	23.4	2.8	1.6	17.6	17.6	.	25.1	10.3
CvsD	7.2	4.2	2.9	24.0	2.2	29.0	.	28.2	2.2
CvsF	10.1	10.0	0.1	34.8	.	.	44.9	0.1	.
CvsH	6.1	3.6	2.5	20.4	16.8	16.8	.	24.0	9.8
CvsI	10.8	6.4	4.4	36.0	.	.	.	42.4	.
DvsF	0.2	2.7	2.9	0.2	2.2	29.3	31.5	28.6	2.2
DvsG	0.3	4.0	4.3	0.3	3.3	42.8	.	41.8	3.3
FvsH	0.2	2.3	2.5	0.2	16.9	16.9	26.8	24.3	9.9
FvsI	0.3	4.1	4.4	0.3	.	.	47.6	43.2	.
GvsH	0.2	3.2	3.4	0.2	23.1	23.1	.	33.2	13.5
**Entire network**	3.8	14.0	2.5	10.7	9.2	17.6	13.0	23.7	5.4
**Included studies**	1	1	1	2	1	2	1	1	1

Interventions are sequenced as follows: A. Oral 400ug , C. Sublingual 400ug , D. Sublingual 600ug , F. Vaginal 200ug , G. Vaginal 400ug , H. Vaginal 600ug , I. Vaginal 800ug.

Supplementary Figure S7. Calculation of the difference between direct and indirect estimates in all closed loops in the analysis of main side effects

Loop	IF	95%CI (truncated)	Loop-specific Heterogeneity(τ^2)
D-H-I	1.36	(0.15,2.56)	0.000
A-G-H-I	1.17	(0.00,3.42)	0.000
A-C-G	0.22	(0.00,1.02)	0.000

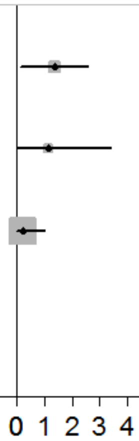

Abbreviations: IF, inconsistency factors.

Interventions are sequenced as follows: A. Oral 400ug , C. Sublingual 400ug , D. Sublingual 600ug , G. Vaginal 400ug , H. Vaginal 600ug , I. Vaginal 800ug.

Supplementary Figure S8. Calculation of the difference between direct and indirect estimates in all closed loops in the analysis of nausea or vomiting

Loop	IF	95%CI (truncated)	Loop-specific Heterogeneity(τ^2)
D-H-I	2.32	(0.15,4.49)	0.000
A-H-I	0.81	(0.00,2.58)	0.000
A-G-I	0.41	(0.00,3.25)	0.000
A-C-G	0.27	(0.00,2.07)	0.000

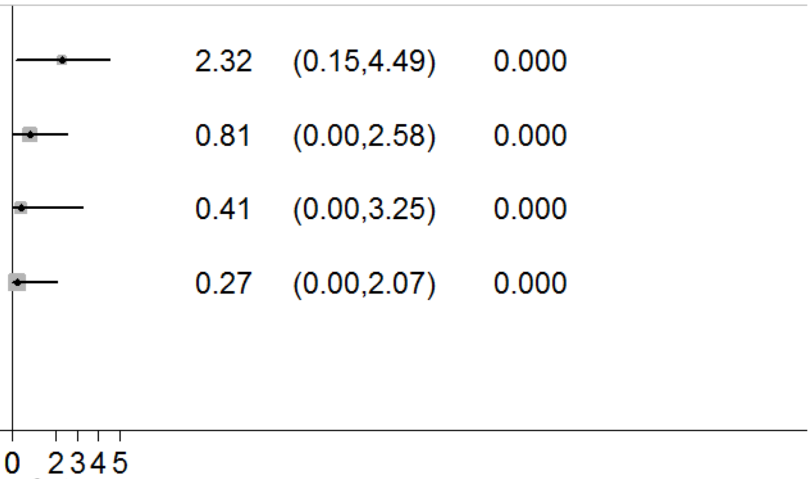

Abbreviations: IF, inconsistency factors.

Interventions are sequenced as follows: A. Oral 400ug , C. Sublingual 400ug , D. Sublingual 600ug , G. Vaginal 400ug , H. Vaginal 600ug , I. Vaginal 800ug.

Supplementary Figure S9. Comparison-adjusted funnel plot in the analysis of main side effects.

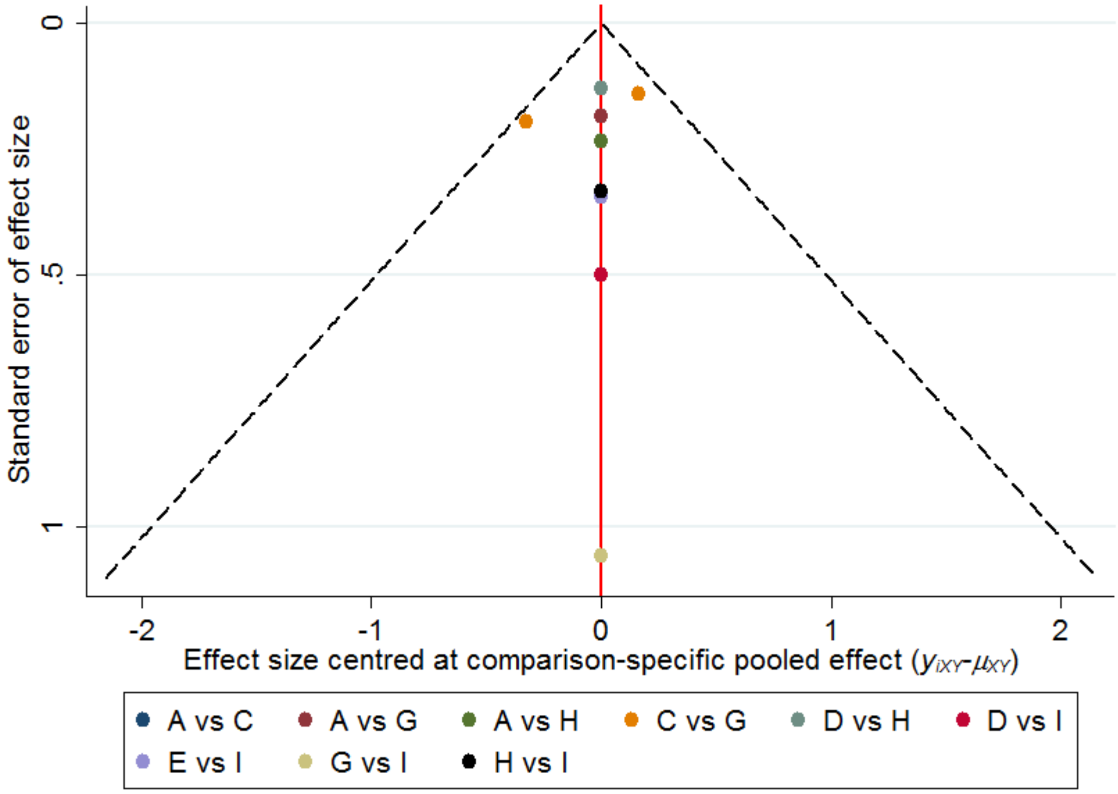

Interventions are sequenced as follows: A. Oral 400ug , C. Sublingual 400ug , D. Sublingual 600ug , E. Sublingual 800ug , G. Vaginal 400ug , H. Vaginal 600ug , I. Vaginal 800ug.
